# Basal Cell Carcinoma in the Periungual Region: A Rare Case and Pathogenesis Insights

**DOI:** 10.7759/cureus.45934

**Published:** 2023-09-25

**Authors:** Jesus Ivan Martinez-Ortega, Felipe de Jesus Perez Hernandez, Leslie Natalia Reyes Garcia

**Affiliations:** 1 Dermatology, Dermatological Institute of Jalisco "Dr. Jose Barba Rubio", Zapopan, MEX; 2 Department of Internal Medicine, High Specialty Regional Hospital of the Yucatan Peninsula, Merida, MEX; 3 Dermatology, Hospital Universitario Clinico Quirurgico Comandante “Manuel Fajardo”, La Habana, CUB

**Keywords:** oncogenesis, periungual, atypical areas, sonic hedgehog, basal cell carcinoma (bcc)

## Abstract

This case report presents a rare occurrence of basal cell carcinoma (BCC) in the periungual region of the thumb. BCC is the most common type of skin cancer, typically found in sun-exposed areas. The discussion explores the underlying pathogenesis mechanisms, including the role of ultraviolet exposure, the absence of pilosebaceous units, and the involvement of the sonic hedgehog (SHH) pathway. Understanding the complexities of BCC in atypical locations is essential for effective prevention and treatment strategies.

## Introduction

Basal cell carcinoma (BCC) is the most prevalent form of cancer globally, and its incidence is on the rise [1]. While BCC commonly occurs in sun-exposed areas, such as the face and neck, its occurrence in the periungual region is rare, with limited reports in the literature [2]. In this case report, we present a unique case of BCC developing in the periungual area of the right thumb and discuss the underlying pathogenic mechanisms contributing to its occurrence in this atypical location.

## Case presentation

**Figure 1:**
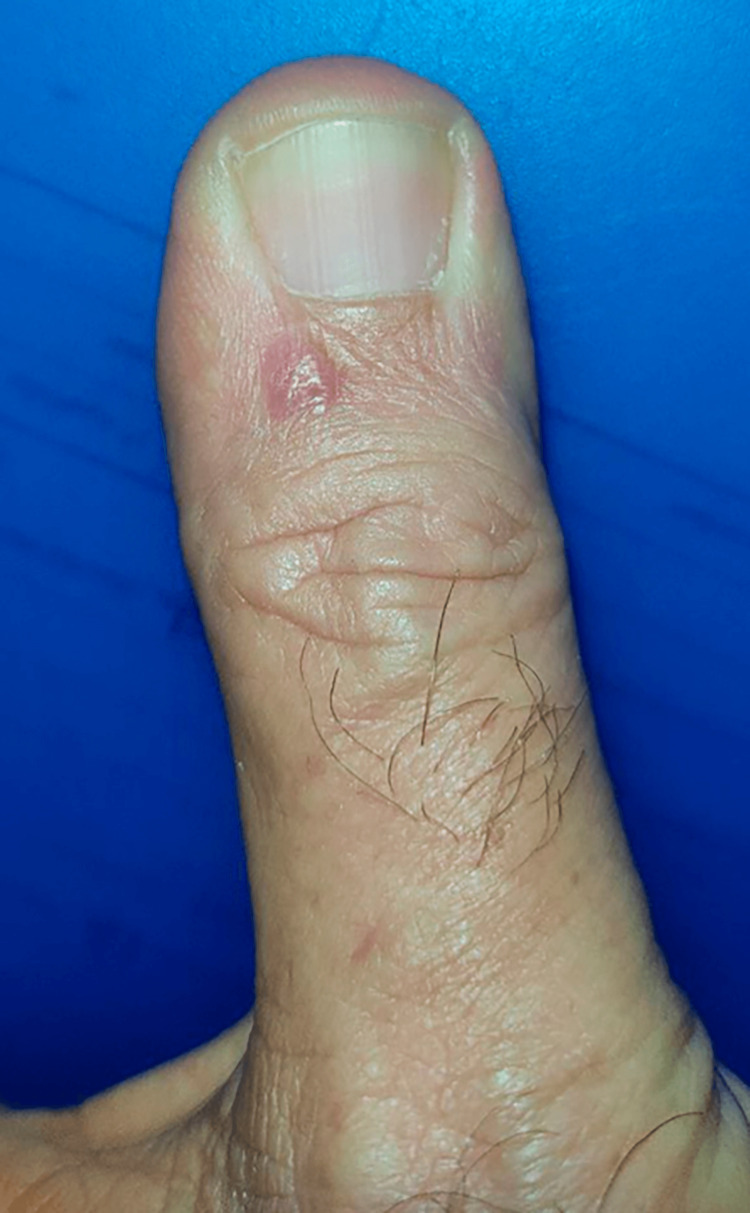
Clinical image A rounded plaque-like growth measuring 8 mm in diameter was observed on the proximal nail fold of the right thumb.

A 54-year-old man with a history of well-controlled hypertension visited with a complaint about a finger lesion that had been present for two years (Figure 1). Upon examination, a rounded plaque-like growth measuring 8 mm in diameter was observed on the proximal nail fold of the right thumb.

**Figure 2:**
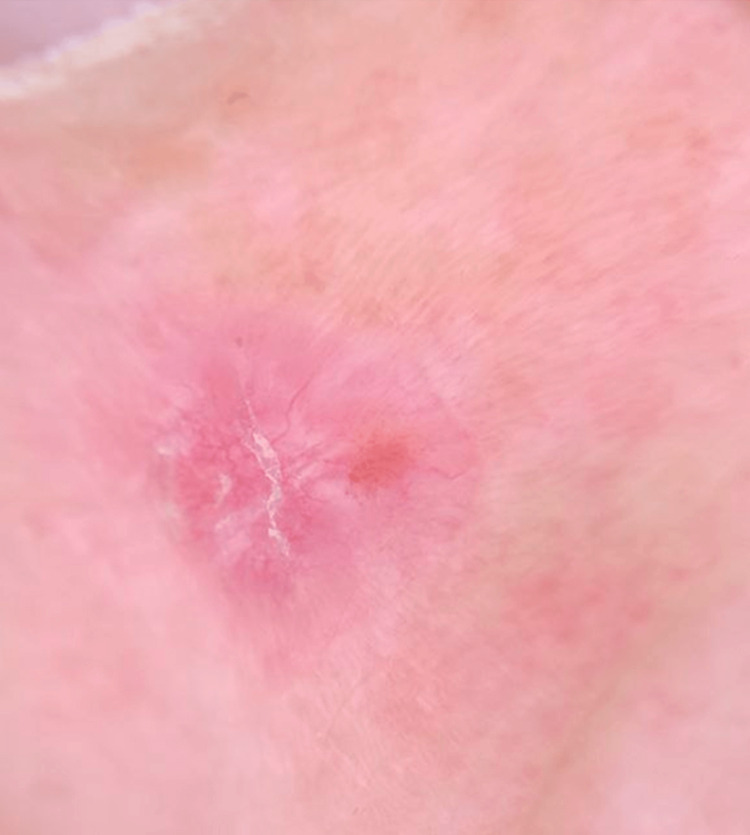
Dermoscopy Dermoscopy revealed characteristic features of a non-melanocytic lesion, including redness, dotted appearance, globules, arborescent vessels, and areas of disrupted white coloration.

Dermoscopy revealed characteristic features of a non-melanocytic lesion, including redness, a dotted appearance, globules, arborescent vessels, and areas of disrupted white coloration (Figure 2).

**Figure 3:**
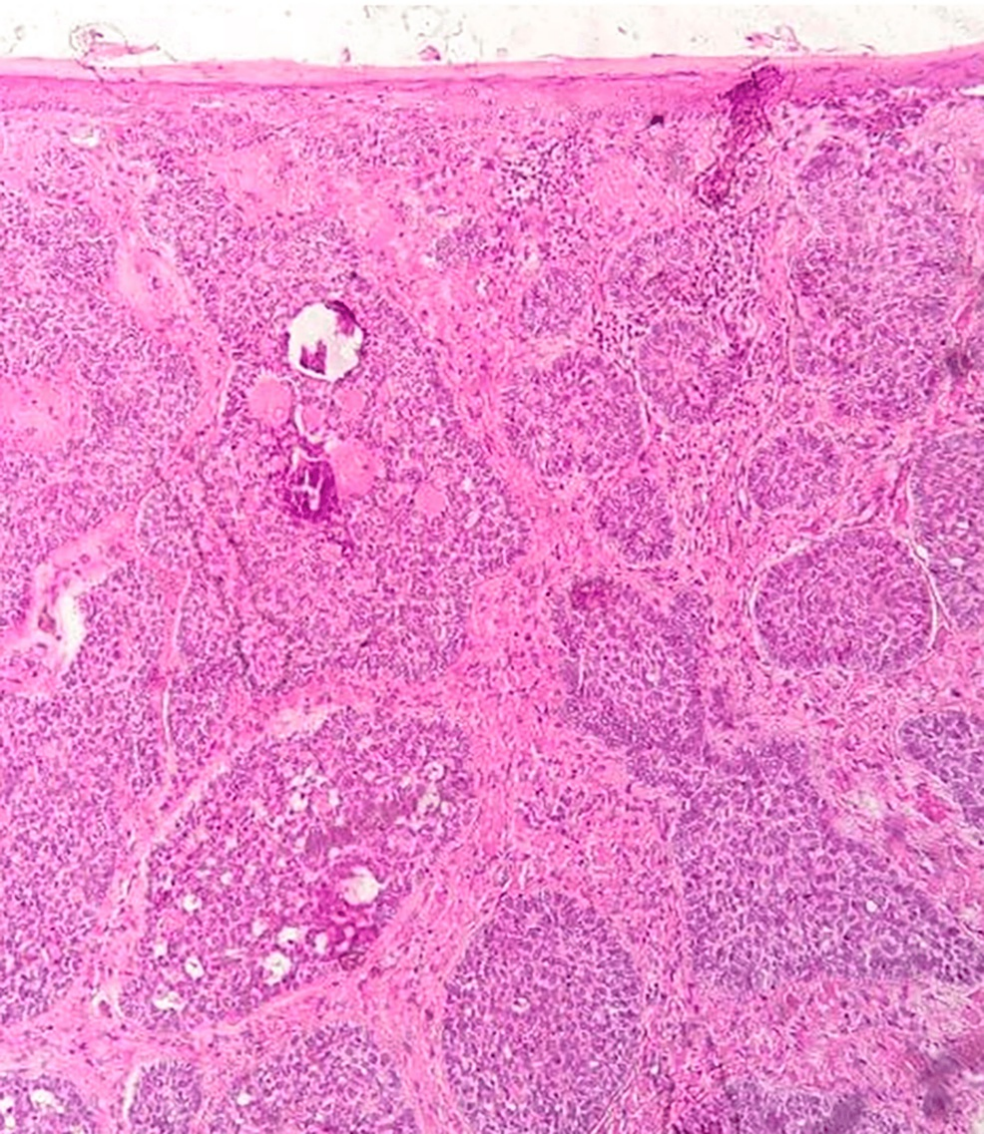
Hematoxylin and eosin stain Hematoxylin and eosin stain depicts basaloid cell nests arranged in a palisading pattern within the top layer of the skin.

Dermatopathologic analysis confirmed the presence of basaloid cell nests arranged in a palisading pattern within the top layer of the skin (Figure 3). Based on these findings, a nodular basal cell carcinoma diagnosis was made, and the lesion was subsequently treated with surgical excision. After a six-month follow-up, there were no signs of a recurrence.

## Discussion

BCC accounts for approximately 80% of all skin cancers, making it the most common type of cancer [1]. Although the hand and upper extremities are not the most common sites for BCC lesions, comprising only about 10% of cases, ultraviolet (UV) exposure is considered the primary cause. The head, neck, and upper extremities are highly exposed to UV radiation, and it is believed to play a crucial role in the development of both BCC and squamous cell carcinoma (SCC) [2].

To explore the existing literature, we conducted a search on PubMed using the terms "basal cell carcinoma" and "nail fold" or “periungual” spanning from 1931 to 2022. Our search yielded 32 cases, with the thumb being the most affected finger. Interestingly, most cases had a disease duration of four to five years, and a significant number had received different initial diagnoses, highlighting the low index of suspicion for BCC in these locations. It is worth noting that most of these misdiagnoses occurred prior to the use of dermoscopy. We believe that the widespread adoption of dermoscopy has facilitated the detection of more cases, even in situations where there is no suspicion of BCC. Also, most of the cases affected fingernails, according to Mortada et al., who conducted a recent systematic review of BCC in the hands and identified 34 cases out of 2,051 patients with involvement of the fingernails, which also underscored the high variability of morphological features [3].

Regarding BCCs occurring in atypical locations, Carter-Grine et al. proposed interesting hypotheses about the underlying oncogenic mechanisms. They suggested that the rarity of BCC in these locations may be associated with the absence of pilosebaceous units, as the oncogenesis of BCC is typically related to hair follicles [4]. However, recent research has shown that BCC can also arise from innervated cells known as touch dome cells and hair follicle stem cell populations (upon deletion of Ptch1) [5]. Given the high innervation of the hands, it is possible to observe BCC in these atypical locations [6]. Moreover, the expression of hedgehog genes during embryogenesis is crucial for the formation of limbs, hair follicles, and peripheral nerves [7]. Furthermore, the density of pilosebaceous units and cutaneous nerves, which are intricately regulated by the nervous system [8], may contribute to the higher incidence of BCC in the head and neck region compared to the upper extremities. It is also worth noting that BCC on the dorsum of the hand shows a similar frequency to other body sites, excluding the face and neck, where BCC is most commonly observed [9]. This suggests that the oncogenesis of BCC is a multifaceted process involving various genetic mutations in the sonic hedgehog (SHH) pathway as well as non-canonical pathways that ultimately converge downstream on the GLI expression [10]. While UV light exposure and the density of pilosebaceous units may partially explain the higher occurrence in the head and neck, it is important to consider that other factors, yet to be fully understood, may activate non-canonical SHH pathways independently of UV exposure. We believe that somatic mutations in cell-intrinsic processes during development may provide some anatomical regions with more proclive for BCC oncogenesis.

## Conclusions

The development of BCC is influenced by multiple factors, including UV exposure, the presence of pilosebaceous units, and intricate mechanisms within and beyond the SHH pathway. Understanding these complex interactions is crucial for comprehending the occurrence of BCC in atypical locations and advancing our strategies for prevention and treatment.

It is conceivable that somatic mutations play a role in influencing these mechanisms in certain individuals, potentially rendering specific anatomical regions more prone to developing BCC at these less common sites. This case encourages us to think beyond the conventional and consider the possibility of BCC at uncommon sites.
